# Attentional Processing in C57BL/6J Mice Exposed to Developmental Vitamin D Deficiency

**DOI:** 10.1371/journal.pone.0035896

**Published:** 2012-04-26

**Authors:** Lauren R. Harms, Karly M. Turner, Darryl W. Eyles, Jared W. Young, John J. McGrath, Thomas H. J. Burne

**Affiliations:** 1 Queensland Brain Institute, The University of Queensland, St Lucia, Queensland, Australia; 2 Queensland Centre for Mental Health Research, The Park Centre for Mental Health, Richlands, Queensland, Australia; 3 Department of Psychiatry, University of California San Diego, La Jolla, California, United Sates of America; 4 Discipline of Psychiatry, The University of Queensland, St Lucia, Queensland, Australia; Chiba University Center for Forensic Mental Health, Japan

## Abstract

Epidemiological evidence suggests that Developmental Vitamin D (DVD) deficiency is associated with an increased risk of schizophrenia. DVD deficiency in mice is associated with altered behaviour, however there has been no detailed investigation of cognitive behaviours in DVD-deficient mice. The aim of this study was to determine the effect of DVD deficiency on a range of cognitive tasks assessing attentional processing in C57BL/6J mice. DVD deficiency was established by feeding female C57BL/6J mice a vitamin D-deficient diet from four weeks of age. After six weeks on the diet, vitamin D-deficient and control females were mated with vitamin D-normal males and upon birth of the pups, all dams were returned to a diet containing vitamin D. The adult offspring were tested on a range of cognitive behavioural tests, including the five-choice serial reaction task (5C-SRT) and five-choice continuous performance test (5C-CPT), as well as latent inhibition using a fear conditioning paradigm. DVD deficiency was not associated with altered attentional performance on the 5C-SRT. In the 5C-CPT DVD-deficient male mice exhibited an impairment in inhibiting repetitive responses by making more perseverative responses, with no changes in premature or false alarm responding. DVD deficiency did not affect the acquisition or retention of cued fear conditioning, nor did it affect the expression of latent inhibition using a fear conditioning paradigm. DVD-deficient mice exhibited no major impairments in any of the cognitive domains tested. However, impairments in perseverative responding in DVD-deficient mice may indicate that these animals have specific alterations in systems governing compulsive or reward-seeking behaviour.

## Introduction

An increasing body of epidemiological evidence supports the hypothesis that maternal vitamin D deficiency is a risk-modifying factor for schizophrenia [1], [2]. In a recent case-control study, those with low levels of vitamin D at birth had a two-fold increase in the risk of developing schizophrenia in later life [3]. Moreover, there is now convincing experimental evidence based on a model in Sprague-Dawley rats that developmental vitamin D (DVD) deficiency is associated with a range of schizophrenia-related neurochemical, neuroanatomical, and behavioural phenotypes [4], [5]. As such, these data support the biological plausibility of the epidemiological link between DVD deficiency and schizophrenia.

To date, most of the animal experimental work related to DVD deficiency has been performed in rat models. More recently, two strains of mice (C57BL/6J and 129X1/SvJ) have been used to examine the effect of DVD deficiency on adult mouse behaviour and it was found that in both strains, DVD deficiency promoted a hyper-explorative phenotype [6]. Furthermore, DVD deficiency was associated with enhanced spontaneous locomotion in 129X1/SvJ, but not C57BL/6J mice [6]. Spontaneous hyperlocomotion has also been observed in DVD-deficient rats several times [7], [8], [9], but hyper-exploration has not been observed in the rat model [8]. We have also shown that DVD deficiency altered brain morphology in C57BL6/J, but not 129X1/SvJ mice, in which adult C57BL/6J male DVD-deficient mice had smaller lateral ventricles compared to controls, which may have been compressed by the enlarged striatum seen in these DVD-deficient mice [10]. This is in contrast to what is seen in DVD-deficient rats, which have *increased* lateral ventricular volume [11], [12]. In addition, DVD-deficient rats were found to be hypersensitive to the locomotor-stimulating effects of the psychotomimetics d-amphetamine and MK-801, indicating possible disruptions in the dopamine and glutamate systems of these animals [9], [13], [14], [15]. Such effects were not seen in DVD-deficient mice [10]. Another study also found that DVD-deficient C57BL/6J mice have subtly impaired performance in an olfactory tubing maze, which is an operant conditioning paradigm requiring mice to learn the associations between a scent and reward [16]. These findings support the hypothesis that there are DVD deficiency-induced effects on behaviourally-relevant neuronal systems. However, little is known regarding how DVD deficiency affects cognitive behaviours that are specifically relevant to schizophrenia.

Cognitive impairments associated with schizophrenia are predictive of functional outcomes [17], [18]. Generally, these impairments are not responsive to antipsychotic drugs [19]. Attention/vigilance is one major cognitive function that is impaired in patients with schizophrenia [20], [21]. Attention is typically measured in patients using the Continuous Performance Test (CPT), and a version of this (CPT-Identical Pairs, IP) has been recommended for use in assessing attention in patients with schizophrenia [22]. The CPT can assess attentional performance by measuring a patient's ability to respond to target stimuli, while withholding their response to non-target (or noise) stimuli. Using signal detection theory, several parameters can be used to assess performance in the CPT. One such example is the discrimination index (d′) that can be used to grade the subject's ability to discriminate between targets and non-targets.

Several tests have now been developed in rodents with the aim of modelling the tests in humans, such as the CPT, which can be used to examine attention. The 5-Choice Serial Reaction Task (5C-SRT) was developed as a test of attention in rats [23]. It has become one of the primary methods used to assess aspects of attentional function in rodents, yet this test does not truly recapitulate the human test (CPT) that is widely used to measure attention. This is because there are no non-target trials in the 5C-SRT in which the animal is expected to withhold their response. Therefore, to more accurately model the human CPT, Young et al. (2009) developed the Five-Choice Continuous Performance Test (5C-CPT) in mice, which was comprised of target and non-target trials. Target trials progress in a similar fashion to trials in the 5C-SRT, requiring a response to a singly lit aperture, but in non-target trials, the whole array of five lights were lit and the mouse was required to withhold a nose poke response to obtain a reward. This method allows the quantification of the parameters such as d′, thus effectively modelling the tests that are used to assess the attentional impairments seen in patients with schizophrenia.

Another aspect of attention that is altered in psychiatric disorders, including schizophrenia, is Latent Inhibition (LI). LI is a learning phenomenon in which pre-exposure to an inconsequential stimulus diminishes future learning of new associations for that stimulus [24]. Patients with schizophrenia, as well as several animal models of risk factors for schizophrenia, exhibit *disrupted* LI, meaning that pre-exposure to a stimulus does not affect leaning new associations for that same stimulus [25]. LI can be measured in rodents in many conditioning paradigms, including fear conditioning and active avoidance. Disruptions in LI were found in DVD-deficient rats [26]. Control rats that were preexposed (PE) to the conditioned stimulus (CS) before training in an active avoidance test exhibited fewer avoidance responses during training compared to controls in the non-preexposed (NPE) group, indicating that they had not learnt the association as well as the NPE group and therefore that LI had occurred in the control population. However, PE DVD-deficient rats performed equally to NPE DVD-deficient rats, indicating that pre-exposure to the CS did not impair subsequent learning (i.e. disrupted LI) [26].

Although DVD-deficient rats have selective cognitive impairments [26], DVD-deficient mice have thus far only been tested in a limited array of cognitive tasks [6], [16] and were found to have subtly impaired performance compared to controls. However, none of the investigations performed in DVD-deficient mice to date directly assessed any of the domains of cognition that are impaired in schizophrenia. It is important to examine the effects of DVD deficiency on cognition in a mouse model because of the wealth of models of genetic risk factors for schizophrenia available in C57BL/6 mice, making the mouse model of DVD deficiency a possible candidate for models combining both genetic and environmental factors. Therefore, the aim of the current study was to further examine learning, memory and cognition in DVD-deficient mice by investigating attention/vigilance using the 5C-SRT and 5C-CPT, and learning, memory and LI using a fear conditioning paradigm.

## Results

### Attention/Vigilance

#### 5C-SRT

**Figure 1:**
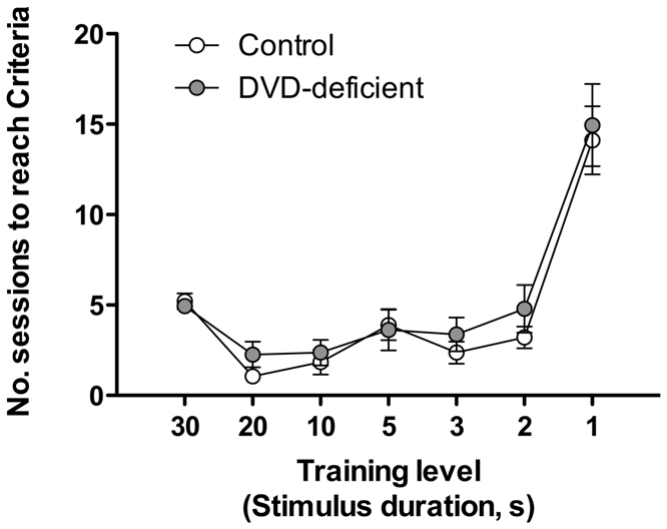
Acquisition of the 5C-SRT. The graphs show the number of sessions (days) to reach criteria for control (*n* = 19) and DVD-deficient (*n* = 19) mice on each of the increasingly-challenging training levels (with stimulus duration decreasing from 30 to 1 s). Data are expressed as mean ± SEM.

**Figure 2:**
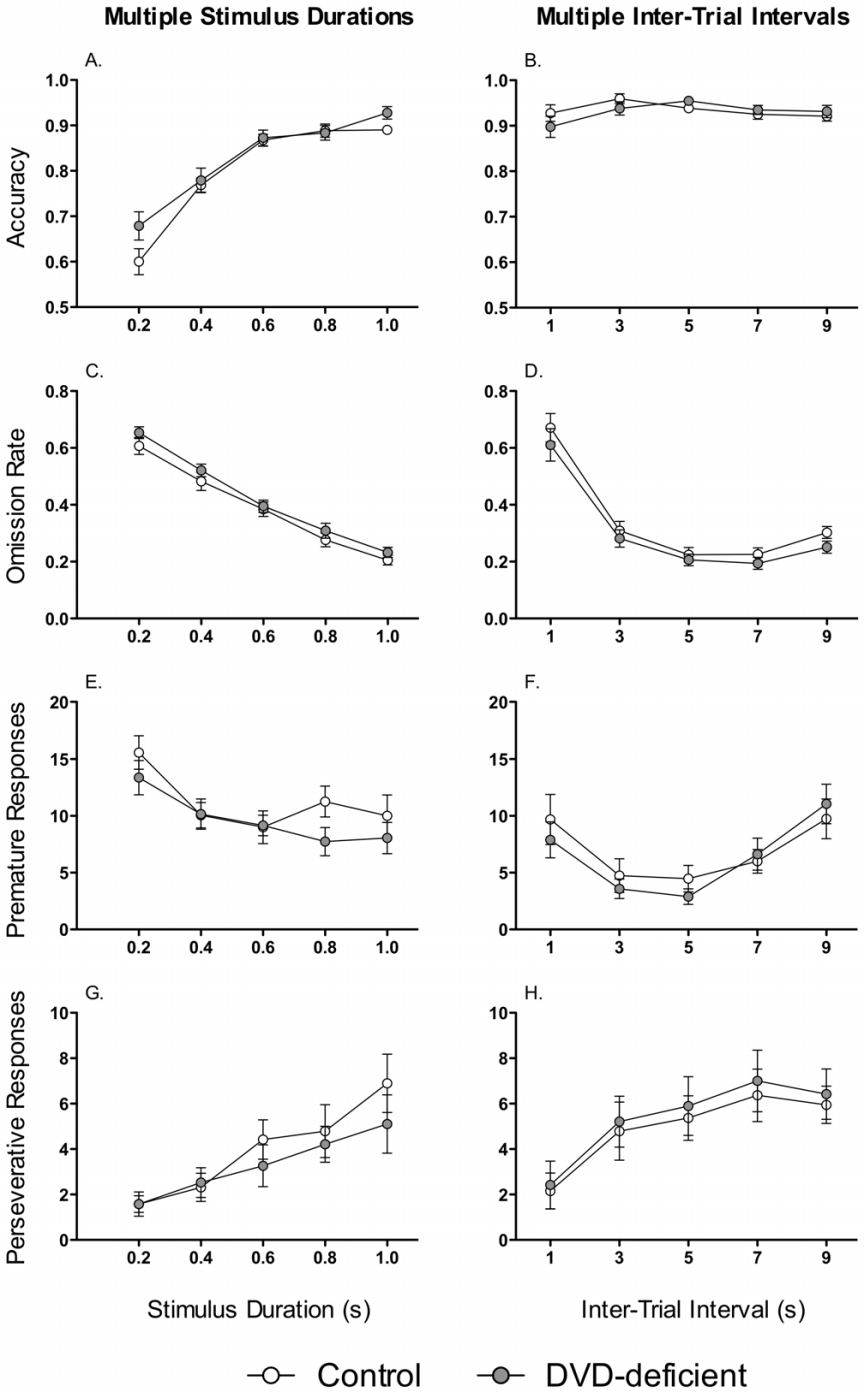
Performance of mice on the 5C-SRT with multiple stimulus durations (left) and multiple inter-trial intervals (right). This figure shows accuracy (**A** and **B**), the omission rate (**C** and **D**), premature responses (**E** and **F**) and perseverative responses (**G** and **H**) for control (*n* = 19) and DVD-deficient (*n* = 19) mice over the five different stimulus durations (0.2–1.0 s) and over the five different inter-trial intervals (1–9 s). Data are expressed as mean ± SEM.

Both control and DVD-deficient mice had similar learning rates in the 5C-SRT training levels (*F*_1,34_ = 2.03, *P* = 0.163; Fig. 1). All mice required the maximum number of testing sessions to reach criteria for level 7 (stimulus duration = 1 s). On the 3-day multiple stimulus durations level, mice were challenged to respond to a range of stimulus durations (0.2–1.0 s). There was no effect of Maternal Diet on any measure of performance in the multiple stimulus durations task, including accuracy (*F*_1,34_ = 3.11, *P* = 0.087; Fig. 2a), omission rate (*F*_1,34_ = 1.20, *P* = 0.281; Fig. 2c), premature responses (*F*_1,34_ = 0.38, *P* = 0.540; Fig. 2e) or perseverative responses (*F*_1,34_ = 1.67, *P* = 0.205; Fig. 2g). At low stimulus durations, all mice made more omissions (*F*_1,34_ = 69.78, *P*<0.001; Fig. 2c) and were less accurate when responding (*F*_1,34_ = 235.04, *P*<0.001; Fig. 2a). On the 3-day multiple inter-trial intervals level, there was no effect of Maternal Diet on performance over various inter-trial intervals on measures such as accuracy (*F*_1,34_ = 0.04, *P* = 0.837; Fig. 2b), omission rate (*F*_1,34_ = 2.59, *P* = 0.117; Fig. 2d), premature responses (*F*_1,34_ = 0.11, *P* = 0.737; Fig. 2f) or perseverative responses (*F*_1,34_ = 0.32, *P* = 0.575; Fig. 2g). Over the different inter-trial intervals, the omission rate was fairly stable, except for an increase in omissions in trials after an inter-trial interval of 1.0 s, (Effect of inter-trial interval *F*_4,34_ = 97.6, *P*<0.002; Fig. 2d).

#### 5C-CPT

**Figure 3:**
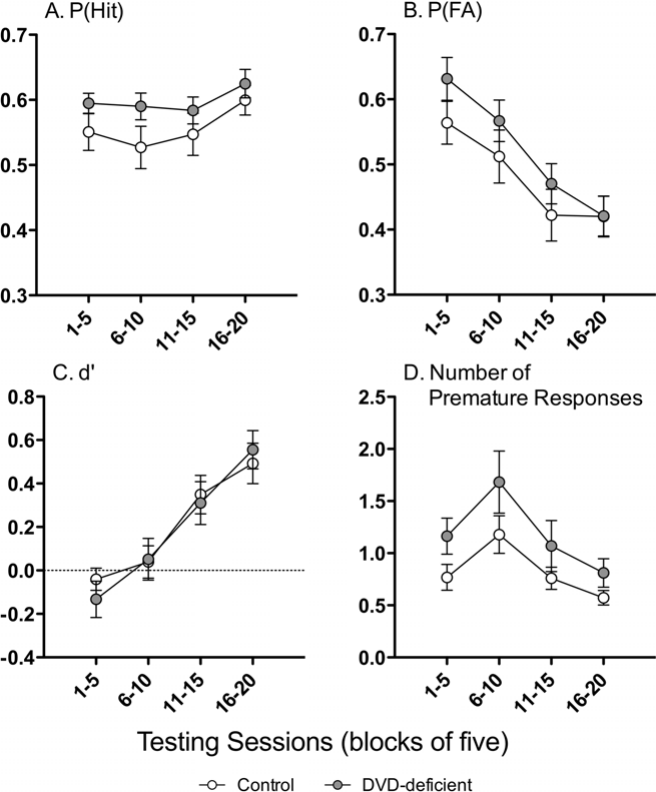
Performance of mice on the 5C-CPT. This figure shows the P(Hit) (**A**), the P(FA) (**B**), the discrimination index d′ (**C**) and premature responses (**D**) for control (*n* = 19) and DVD-deficient (*n* = 19) mice averaged over 4 blocks of 5 trials. DVD-deficient mice also made more premature responses than control mice, although this did not reach significance (Main effect of Diet *F*_1,34_ = 3.66, *P* = 0.064). Data are expressed as mean ± SEM. * *P*<0.05 and τ *P*<0.1 between control and DVD-deficient mice.

Over the 20-session 5C-CPT test, DVD-deficient mice had similar levels of performance to controls on the primary measures of performance; P(Hit) (*F*_1,34_ = 2.01, *P* = 0.165; Fig. 3a), P(FA) (*F*_1,34_ = 1.57, *P* = 0.219; Fig. 3b), and d′ (*F*_1,34_ = 0.05, *P* = 0.830; Fig. 3c). DVD-deficient mice appeared to make more premature responses than control mice, although this did not reach significance (Main effect of Diet *F*_1,34_ = 3.66, *P* = 0.064; Fig. 3d).

**Figure 4:**
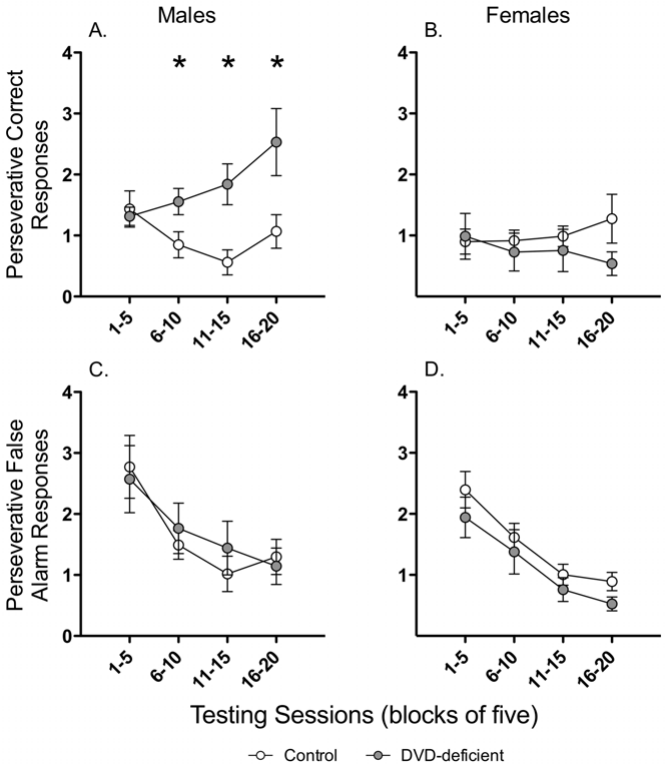
Perseverative responding in the 5C-CPT. This figure shows the perseverative correct responses (**A** and **B**) and the perseverative false alarm responses (**C** and **D**) for control (*n* = 9–10) and DVD-deficient (*n* = 9–10) males (left) and females (right) averaged over 4 blocks of 5 trials. DVD-deficient males made more perseverative responses during target trials, particularly in the later sessions (Sessions 6–10 *t*_1,17_ = −2.32, *P* = 0.033; Sessions 11–15 *t*_1,17_ = −3.16, *P* = 0.006; Sessions 16–20 *t*_1,17_ = −2.31, *P* = 0.034). Data are expressed as mean ± SEM. * *P*<0.05 and τ *P*<0.1 between control and DVD-deficient mice.

DVD-deficient males made more perseverative responses during target trials (Sex×Diet interaction *F*_1,34_ = 6.42, *P* = 0.016; Fig. 4a), particularly in the later sessions (Sessions 6–10 *t*_1,17_ = −2.32, *P* = 0.033; Sessions 11–15 *t*_1,17_ = −3.16, *P* = 0.006; Sessions 16–20 *t*_1,17_ = −2.31, *P* = 0.034). This effect on perseverative responses was restricted to the correct responses during target trials, not the false alarm responses in non-target trials, for which there was no significant effect of Maternal Diet (*F*_1,34_ = 2.50, *P* = 0.123; Fig. 4c,d).

### Fear conditioning

**Figure 5:**
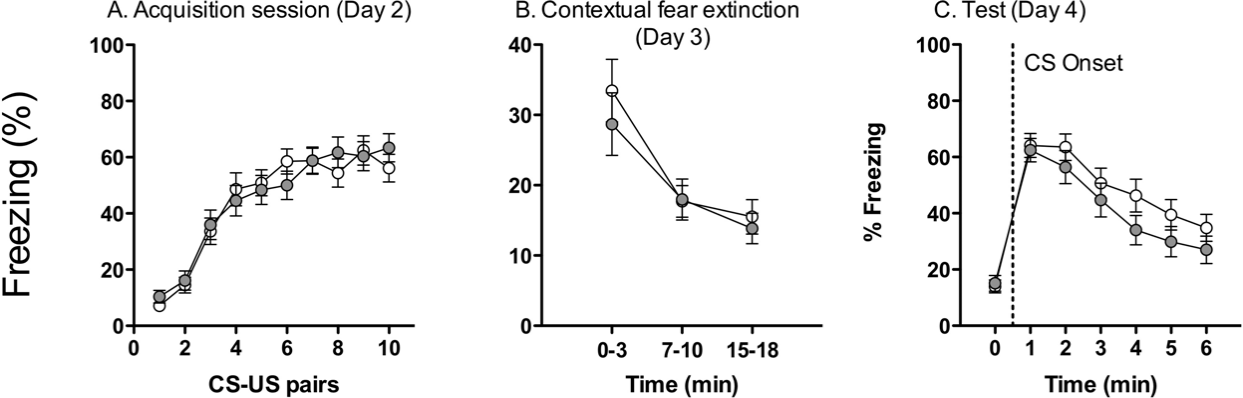
Acquisition, contextual fear extinction and cued test of conditioned fear. Data show the freezing scores (%) over the 10×30 s CS presentations in the acquisition session (Day 2; **A**), the 3×3 min sampling intervals in the context extinction session (Day 3; **B**) and for the 3 min interval prior to CS onset, followed by the 6×1 min time bins after CS onset in the retention test session (Day 4; **C**) for control (*n* = 31) and DVD-deficient (*n* = 31) mice in the NPE group. Data are expressed as mean ± SEM.

To exclusively assess fear conditioning in DVD-deficient mice, a separate analysis of mice in the NPE group was performed. There was no effect of Maternal Diet on the acquisition of the association between the CS and the US, because DVD-deficient mice exhibited similar levels of conditioned freezing to control mice on day two of testing (*F*_1,58_ = 0.24, *P* = 0.628; Fig. 5a). All mice exhibited an increase in CS-evoked freezing throughout the session (effect of CS number *F*_1,58_ = 55.18, *P*<0.05).

On day 3 of testing, DVD deficiency was not associated with a change in the extinction of contextual fear (*F*_1,58_ = 0.10, *P* = 0.757). All groups exhibited the predicted decrease in freezing over the testing period (effect of time *F*_1,58_ = 17.95, *P*<0.01; Fig. 5b). On day 4 of testing, all mice displayed retention of conditioned fear as shown by the increase in freezing responses upon CS onset (effect of Time *F*_1,58_ = 65.69, *P*<0.05; Fig. 5c), but maternal diet was not associated with an alteration in the expression of conditioned fear for the CS (*F*_1,58_ = 0.59, *P* = 0.446).

### Latent Inhibition

**Figure 6:**
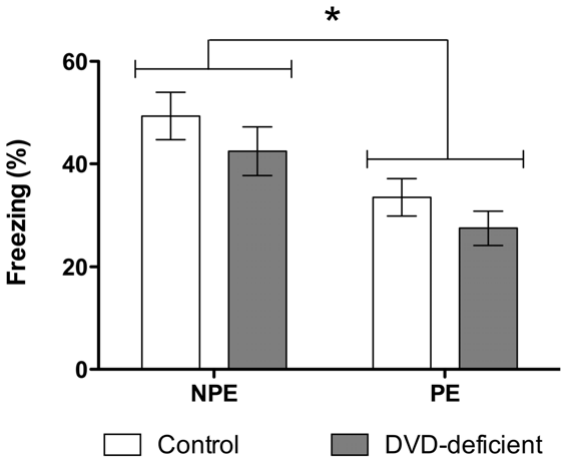
LI in the retention test session (Day 4). Data show the freezing scores (%) for control and DVD-deficient mice in the NPE (n = 31) and PE (n = 30) groups. Mice in the PE groups exhibit less conditioned freezing than NPE mice (Main effect of Pre-exposure *F*_1,114_ = 13.03, *P*<0.001), indicating LI (the inhibition of learning caused by pre-exposure to the CS). Data are expressed as mean ± SEM. * *P*<0.05 between NPE and PE mice.

LI was examined on day 4 of testing, and was evident as a decrease in conditioned fear in PE mice (*F*_1,114_ = 13.03, *P*<0.001; Fig. 6). Post-hoc *t* tests indicated that LI was present in both control (*t*_1,59_ = 2.69, *P* = 0.009) and DVD-deficient mice (*t*_1,59_ = 2.59, *P* = 0.012). Furthermore, DVD-deficient mice did not exhibit an altered LI response, as there was no significant Maternal Diet×Pre-Exposure interaction (*F*_1,114_ = 0.01, *P* = 0.907).

### Nociception

The hot plate and tail flick tests revealed that DVD-deficient mice were similar to control mice in their latencies to remove their paws/tail from painful stimuli. In the hot plate test, control mice had a latency of 18.99±0.46 s to remove their paws from the heat source and DVD-deficient mice a latency of 18.17±0.59 s (*F*_1,118_ = 1.33, *P* = 0.251). For the tail flick test, mice had latencies of 4.09±0.13 s and 4.03±0.12 s to move their tails for the control and DVD-deficient groups, respectively (*F*_1,118_ = 0.33, *P* = 0.565).

## Discussion

This study presents the first characterization of the consequences of DVD deficiency on attentional processing in adult mice. We found that DVD deficiency in mice was associated with subtle alterations in performance on the 5C-CPT, independent from any changes in overall performance in the domains of attention, learning, memory and LI.

Responses on the 5C-SRT revealed that sustained and selective attention in mice was largely unaltered by DVD deficiency when the mice were presented with only target stimuli. Both control and DVD-deficient mice learned the 5C-SRT and progressed through the training levels at similar rates. Moreover, when challenged with manipulations in which the stimulus duration and inter-trial interval were varied, DVD-deficient mice performed similarly to controls. At low stimulus durations and with increased attentional load, all mice made more omissions, and when they did respond, had lower accuracy, consistent with previously published findings in the 5C-SRT [27], [28]. In the inter-trial interval manipulations there was a predictable effect of inter-trial interval on premature responses, whereby all mice made more premature responses at longer inter-trial intervals.

In order to investigate aspects of vigilance and *selective* attention in DVD-deficient mice, the 5C-CPT was used. In the 5C-CPT experiments, both DVD-deficient and control mice began the sessions with their d′ level at approximately 0, indicating that mice were not discriminating between the target and non-target trials. Throughout the sessions, however, the d′ improved in all groups and by session 20, was at least 0.50 in all groups, indicating that mice were indeed responding appropriately in both target and non-target trials. DVD-deficient mice exhibited similar P(Hit) and P(FA) levels to control mice throughout the 20 sessions and had similar d′ levels, indicating that discrimination was not affected by DVD deficiency when the fundamental measures of signal detection were investigated.

There were, however, some subtle effects of DVD deficiency on performance in the 5C-CPT. DVD-deficient male mice made more perseverative responses in the CPT, but not during the early sessions (1–5). In target trials, a perseverative response occurred when a mouse had already made a correct nose poke and continued to make repeated nose pokes before collecting their reward. In non-target trials, perseverative responses occurred after a mouse had made a false alarm response and made more nose pokes during the time out period. Therefore, perseverative responses on target and non-target trials represent slightly different responses: one is the repetition of a correct response that leads to reward, and the other is the repetition of an incorrect response that leads to a time out or punishment. By definition, perseverative responses occur after the mouse has made the response that ends the trial (either with a reward or a time out) and therefore are not rewarded or punished.

Increased perseverative responses may indicate that DVD-deficient mice have a response inhibition deficit. However, DVD-deficient mice had normal response inhibition in other elements of the task; they did not make more false alarms, although there was a non-significant trend towards an increase in the amount of premature responses in DVD-deficient mice. Although both perseverative and premature responses represent a lack of inhibition, there are several differences between the two. Premature responses represent motoric impulsivity, or the inability to wait and to withhold a planned response. On the other hand, perseverative responses may represent compulsive behaviour, or the inability to inhibit the repetition of actions. Further evidence suggests that impairments in inhibition with regard to premature (impulsive) responses are neurobiologically separate from perseverative (compulsive) responses [29]. There is some evidence to suggest that premature and false alarm responses are governed by separate systems [30] and the current study adds to this body of knowledge by further divorcing perseverative correct responses from false alarm and perseverative false alarm responses, indicating that the 5C-CPT is a sensitive test that can be used to discriminate between very specific types of response inhibition. This is an important consideration for animal models of neuropsychiatric disorders because of the shift towards investigating individual endophenotypes of disorders (e.g. compulsivity or impulsivity) as opposed to entire disorders (e.g. attention deficit hyperactivity disorder or obsessive-compulsive disorder) [31].

The changes in perseverative responding, if indicative of a phenotype of repetitive or compulsive behaviour, could be interpreted within the context of previous findings from studies in DVD-deficient mice, in which C57BL/6J mice exposed to DVD deficiency were hyper-explorative on the hole board test, head dipping more over the two-day test than their control counterparts. This behaviour may indicate a hyper-responsiveness to rewarding stimuli. After initial neophobia, exploration of a novel environment is highly rewarding for a rodent [32]. In the 5C-CPT, DVD-deficient mice only display increased responses that they have learnt to be associated with a reward (perseverative correct responses). Taken together, previous findings of enhanced exploration and those here of a perseverative reward-associated response, may suggest a reward-seeking phenotype in these animals. Further study could be aimed at probing the function of the reward system in these animals, perhaps by investigating free-choice drug seeking behaviours or by examining place-preference for drugs of abuse. However, such a reward-seeking phenotype would typically be associated with other behaviours that have not been observed in DVD-deficient C57BL6/J mice, namely, spontaneous hyperlocomotion and hypersensitivity to amphetamine [32].

An alternative explanation for the increased head dipping and perseverative responding seen in DVD-deficient mice may be that these behaviours are not related to reward, but may reflect the repetitive and compulsive behaviours commonly associated with obsessive-compulsive disorder (OCD) and autism. Such an association is strengthened by the possible link between the behavioural changes identified in the current study, and morphological changes found in previous studies. Firstly, both the behavioural and morphological alterations were only identified in male, not female mice [10], [16]. Secondly, the neuroanatomical changes seen in DVD-deficient mice have been associated with repetitive behaviour in clinical populations. A reduction in the lateral ventricles was identified in DVD-deficient male mice [10], [16], which has also been found in patients with autism [33]. This reduction in ventricular volume in DVD-deficient mice may have been due to an enlargement of the striatum [10]. Not only have increases in caudate volume been identified in populations with autism [34], but increases in the volume of the right caudate nucleus were associated with the severity of repetitive, OCD-like behaviours in patients [35]. This is also in agreement with evidence that the size of the striatum is increased in patients with OCD [36], [37]. The changes in striatal volume seen in DVD-deficient males may therefore indicate a mechanism underpinning the increases in repetitive behaviour seen in these mice. Further exploration of this phenotype could be directed at assaying other repetitive behaviours in the model, such as barbering, marble burying or reversal learning (the range of OCD-associated behaviours and models is reviewed elsewhere [38]). However, it has already been found that DVD deficiency in mice did not affect levels of social exploration in the social interaction test, nor did it affect open arm exploration on the elevated plus maze [6], indicating that the DVD-deficient mouse, while perhaps representing a model of OCD- and autism-like repetitive behaviours, may not model other aspects of autism and OCD, such as social interaction impairments and anxiety-like behaviour. There is, however, some emerging evidence that DVD deficiency may also be a risk factor for autism. There are as yet no epidemiological studies demonstrating a direct association between autistic spectrum disorders and developmental vitamin D levels, yet there are effects of season of birth, latitude of birth, and migrant status that may indicate a possible association (for review, [39]). If further evidence continues to support this association, the DVD-deficient mouse model may be utilized to further examine the role vitamin D plays in brain development with relevance to autism.

The findings from the remainder of the study indicate that DVD deficiency in mice was not associated with alterations in fear conditioning, LI or nociception. LI was demonstrated in both control and DVD-deficient mice, where the PE group expressed less conditioned freezing during the CS than mice in the NPE group in the testing session. Disruptions in LI were previously found in DVD-deficient rats [26], in conjunction with several other behavioural and neuroanatomical alterations relevant to schizophrenia [8], [9], [11], [12], [13], [14], which have not been observed in the mouse model [6], [10], [16]. Species differences in the response to DVD deficiency have been previously reported in regards to the novel effects on exploratory behaviour [6], which were not seen in DVD-deficient rats [8]. Furthermore, whereas we have reported *increased* ventricular volume in DVD-deficient rats [11], [12] the DVD-deficient mouse has a reduction in ventricular volume. These differences may not necessarily be due to species differences, but may be due to the strain selection. DVD deficiency has been examined in one outbred rat strain and two inbred mouse strains, and perhaps the two strains chosen (C57BL/6J and 129X1/SvJ) may be largely unresponsive to changes in vitamin D levels. Such differences highlight the importance of species and strain selection in studies of altered vitamin D signalling and brain development.

Taken together these findings indicate that attentional processing is largely intact in a C57Bl/6 mouse model of prenatal vitamin D deficiency, suggesting that DVD deficiency has little to no effect on the systems governing attention in the mouse. Nevertheless, utilizing a mouse model of DVD-deficiency leaves open the possibility to combine the model of an environmental risk factor for schizophrenia with one of the numerous models of genetic risk factors available in C57Bl/6 mice. Perhaps such gene×environment models will reveal that a combination of factors results in cognitive impairments associated with schizophrenia.

## Materials and Methods

### Ethics Statement

All procedures were performed with the approval of The University of Queensland Animal Ethics Committee, under the guidelines of the National Health and Medical Research Council of Australia.

### Animals and Housing

A total of 160 C57BL/6J mice were used for the study. Mice were housed in individually ventilated OptiMice cages (Animal Care Systems, CO, USA), at a stable temperature of 21±1°C. For the 5C-SRT and 5C-CPT experiments, 19 males (nine control and 10 DVD-deficient) and 19 females (10 control and nine DVD-deficient) were used from approximately 20 weeks of age. The remainder of the mice were used in the fear conditioning, LI and nociception experiments. These mice were divided into two groups – PE and NPE, resulting in *n* values of 20–22 per maternal diet for males and 9–11 per maternal diet for females. The discrepancy in sample sizes between males and females was due to a low number of female mice being born at those particular times. However, on average, the breeding program generated similar amounts of male and female offspring. Mice were reared, housed and tested at the Queensland Brain Institute animal facility, The University of Queensland. Unless otherwise indicated (for the 5C-SRT and 5C-CPT), mice had free access to food and water throughout testing.

DVD-deficient and control offspring were generated as previously described [6]. Briefly, female mice were fed on a diet free of vitamin D (Vitamin D Deficient AIN93G Rodent diet, Specialty Feeds, WA, Australia) from 4 weeks of age. Control animals were housed in the same conditions as vitamin D-deficient animals, but were given a standard vitamin D-containing diet (Standard AIN93G Rodent diet, Specialty Feeds, WA, Australia). At 10 weeks of age, all females were mated with vitamin D normal males by pairing one male with 2 females for 7 days. Dams remained on these respective diets throughout gestation. Pregnant dams were checked every 12 h for pups and the food of dams on the vitamin D-deplete diet was replaced with control food within 12 h of birth. Offspring were weaned 21 days after birth, and were provided with water and standard vitamin D containing mouse pellets (Feeder and Grower diet, Specialty Feeds, WA, Australia) for the remainder of the experiment. After weaning, control and DVD-deficient offspring were re-identified to conceal the experimental group and all behavioural testing and data processing were performed blind to maternal diet.

### Attention/Vigilance

#### Food Restriction

Beginning from 19–21 weeks of age, one week before the commencement of training, mice were placed on a restricted feeding schedule to decrease body weight to ∼90% of their original free-feeding weight. Mice were weighed, then each box of mice was given 0.075 g of standard feed/g of starting weight/mouse/day at approximately 1600 h. Mice were usually housed in pairs unless their weight loss was uneven, suggesting that one mouse was eating the majority of the food. In these cases, mice were separated until their weight had stabilized at 90%. After 5 days of food restriction, the daily amount of food was increased to 0.1 g food/g free-feeding body weight. Mice were weighed each day prior to testing and food amounts were titrated based on their weight, so they remained at ∼90% free-feeding weight without falling lower than 85%. Mice had free access to water in the home cage throughout testing. On the 5 days of food restriction 1 ml of liquid reinforcer (Breaka strawberry milk, Parmalat, QLD, Australia) was provided to each box of two mice to habituate them to the reinforcer.

#### Testing Apparatus

All training and testing took place in a bank of four nine-hole nose poke mouse operant chambers (Med Associates, VT, USA). However, only five of the nine holes were used in experiments (the first, third, fifth, seventh and ninth holes). Each nine-hole chamber was placed within a larger sound attenuated cabinet (inner dimensions 60×35×56 cm high) so that sound and light exposure could be minimized in the testing environment. The chambers were controlled by a central interface device, which was connected to a computer running MED-PC IV. MED-PC IV was used to run MedState notation programming (.mpc files), which can be used to define and initiate experiment protocols. For all experiments, modified versions of the .mpc file for a standard nine-choice serial reaction time task supplied with the system were used.

#### 5C-SRT

Mice were trained to perform the 5C-SRT according to a standard protocol [40]. Each mouse was trained or tested on one session per day for 5–7 days/week. To start each testing session, the reward was raised into the reward magazine and the reward light was left on. When the mouse head dipped into the reward magazine to drink the reinforcer, the session began. In the first training level (0), lights did not appear in the nose poke holes and mice needed to poke into any hole to retrieve their reward, which was signalled by the reward light. This trial was run for 30 min. Mice needed to receive 70 rewards in one session to proceed to the next phase of testing.

For the next level of testing (level 1), mice had to respond to a light randomly presented in one of five holes with a nose poke in the lit hole to receive a reward. These were target trials, where the mouse had to respond to receive a reward. For all training phases, the time between each trial (the inter-trial interval) was set at 5 s and the stimulus duration began at 30 s for level 1. If a mouse did not respond correctly during the stimulus duration, this was scored as an “omission” and the mouse received a “time out” (i.e. house light on, no stimulus lights, no reward) for 5 s before the commencement of the next trial. The mouse also received a time out if they nose poked into an unlit hole (incorrect response), or if they nose poked during the inter-trial interval (premature response). If a mouse made more than one nose poke response in a correct hole, this was counted as a perseverative response and was also recorded. To reach criteria for the next level of training, a mouse needed to perform <20% omissions and have an accuracy >80% (accuracy was calculated as the percentage of correct responses/total number of responses). Training levels 2–7 proceeded in much the same way as level 1, with the stimulus duration decreasing to 1 s in level 7. The time the mouse had to make a response also decreased with the stimulus duration, but remained at 5 s in the last training levels, even when the stimulus was only present for 1 s.

Once mice had passed criteria for level 7 on 3 days, they were tested for their response to multiple stimulus durations (level 8) in which the stimulus duration varied randomly at values of 0.2, 0.4, 0.6, 0.8 and 1 s (all previous trials were 1 s). All mice were tested for 3 days on multiple stimulus durations regardless of their performance. After another two sessions of baseline (level 7), mice were then tested for 3 days for their response to multiple inter-trial intervals (level 9), which varied randomly at values of 1, 3, 5, 7 and 9 s. Their baseline responses were tested again for another 2 days on level 7.

#### 5C-CPT

After the last 2 days of level 7 (after multiple inter-trial intervals), sessions were altered so that the number of trials was increased to 120 and the inter-trial intervals were variable at 3, 4, 5, 6 and 7 s at level 10. Once a mouse had passed criteria for level 10 (omissions <25%, accuracy >80%), they were advanced to the 5C-CPT (level 11). In the 5C-CPT, 80 of the 120 trials were target trials and were the same as level 10 (with a variable inter-trial interval). The other 40 trials were non-target trials and in order to receive a reward, the mouse needed to withhold a response. For non-target trials, all nine lights were turned on for 1 s, and the mouse had to withhold a nose poke response for 3 s in order to receive a reward. If the mouse made a nose poke response within the 3 s, this was counted as a “false alarm” and they received a 5 s time out. In a similar fashion to the 5C-SRT, nose pokes during the inter-trial interval were punished with a time out. In addition to the perseverative responses after a correct response in a *go* trial, perseverative false alarms were also recorded. Mice were tested for 20 consecutive days on the 5C-CPT regardless of performance.

#### Dependent Variables

Learning rates in the operant 5C-SRT and 5C-CPT tasks were examined by comparing the number of sessions it took the mice to reach criteria for each level. The dependent variables for these tests and how they were calculated are outlined in Table 1. The sessions the 5C-SRT were exclusively comprised of target trials in which accuracy, P(Hit), omission rate, number of premature responses, number of perseverative responses and the latencies to make a correct response, an incorrect response and to collect reward were used to analyse the performance of mice. Dependent variables from the multiple stimulus durations level were averaged over the three testing sessions for each mouse and pooled based on stimulus duration. The same procedure was performed for dependent variables from the multiple inter-trial intervals level, which were pooled based on inter-trial interval.

**Table 1:** Descriptions of the dependent variables used for the analysis of the 5C-SRT and 5C-CPT and how they were calculated.

*Measure* (Target *Trials*)	
*Accuracy*	No. of correct responsesNo. correct responses+no. incorrect responses
*P(Hit)*	No. of correct responses
*Probability of a hit response*	Total no. of target trials
*Omission Rate*	No. of omissionsTotal no. of target trials
*Premature Responses*	No. of premature responses made
*Perseverative Responses (Target trials)*	No. of perseverative correct responses made during target trials
A perseverative response occurred when a mouse nose-poked more than once in the correct hole	
*Latency to Correct Response (s)*	Average time between target stimulus onset and correct nose poke
*Latency to Incorrect Response (s)*	Average time between target stimulus onset and incorrect nose poke
*Latency to Retrieve Reward (s)*	Average time between correct nose poke and reward retrieval

*Measure (Non-target Trials)*	
*P(FA)*	No. of false alarm responses
*Probability of a false alarm response*	Total no. of *non-target* trials
*Perseverative False Alarm (non-target trials)*	No. of perseverative false alarm responses made during *non-target* trials
*In contrast to perseverative responses in target trials, a perseverative response during a non-target trial occurred when a mouse made more than one false alarm nose poke*	
*Latency to False Alarm (s)*	Average time between *non-target* stimulus onset and false alarm nose poke
*d′*	Z score of P(Hit) – Z score of P(FA)
Discrimination Index	

One-third of the trials for the 5C-CPT study, were non-target trials, and therefore for this study, the variables P(FA), number of perseverative false alarms, latency to make a false alarm response and latency to retrieve a reward for a correctly withheld response were added to the analysis with the other variables for target trials. Furthermore, the P(Hit) of target trials and the P(FA) of non-target trials were also used to calculate the discrimination index, d′.

### Fear Conditioning and LI

Mice were tested over 4 consecutive days (one test/day) using a Med Associates (VT, USA) fear conditioning system (Med-VFC-MS). Four Med Associates fear conditioning chambers (ENV-008 FC) were used, which had inner dimensions of 30×25×20 cm high. Each of the chambers had a grid floor (ENV-005 QD) attached to an electric shock stimulator/scrambler (ENV-414S) to deliver the US and a speaker attached to a programmable tone generator to deliver the CS. A fluorescent light (PHM-258-X) was in the top of the chamber, and was left on throughout experiments. The whole chamber sat within a larger sound attenuating cubicle (inner dimensions 60×70×40 cm high), which had a small video camera attached to the door (VDO-CAM-4.65). When the door was closed, the chamber (and the mouse inside) could be viewed using the camera, the output of which was used by the Med Associates software, Video Freeze. The software used the video file to calculate the motion index, the number of pixels that had changed within a designated time period more than they would change if the mouse was not present (i.e., video noise) [41]. Freezing was measured every 2 s and anything under a motion threshold of 20 arbitrary units was regarded as freezing (10–20 is recommended). For each time bin tested, Video Freeze returned a percentage freezing value, which was the percentage of the number of freezing episodes out of the total number of freezing episodes possible in the time bin (30/min). Chambers were cleaned with 70% ethanol after each test.

The LI training and testing protocol was performed similarly to that described in [42]. On the first day of testing, mice were placed in the chamber for an initial 5 min habituation period. For mice in the PE group, the program for day one consisted of two 3-min sessions without any stimuli at the beginning and end, with 40×10 s presentations of a 9 KHz tone, which were pseudo-randomly timed, so that the time between tones was no less than 30 s and no more than 90 s. The program ran for 52 min. NPE mice were put in the chambers, but received no stimulus presentations over the 52 min trial.

From the second day of testing, the programs for PE and NPE mice were identical. Mice were placed in chambers and left to habituate for 5 min before the program and recording began. Again, the first 3 min of the program were stimulus-free, after which, 10×30 s 9 KHz tones (CS) were presented 1 min apart, each immediately followed by a 1 s duration 0.4 mA foot shock (US). After the CS/US pairings, recording continued for another 3 min stimulus-free period. The current protocol did not use different contexts for the training and test periods. Therefore, to minimize contextual fear conditioning, mice were placed in the chamber for 18 min without any tone or shock presentations on the third day of testing.

On day 4, mice were placed in the chamber for 12 min. After 3 min of baseline, stimulus-free recording, the CS (9 KHz tone) was played continuously for 6 min and followed by another 3 min baseline recording period. The dependent variables used for analysis of fear conditioning and LI were percentage freezing levels during CS presentation on the acquisition day (day 2), throughout the trial to extinguish contextual fear (day 3) and before and during the CS test on day 4 in just the NPE mice, in order to assess learning and memory independent of pre-exposure. LI was examined by comparing the freezing levels in NPE and PE on the CS test day (day 4).

### Nociception

Approximately 5 days after the final day of testing, all mice tested in the fear conditioning/LI experiments were tested for their responses to painful stimuli using the hot plate and tail flick tests. A standard hot plate (Harvard Instruments, MA, USA) was heated to 55°C for 10 min prior to testing, and a tall Perspex cylinder was placed on the plate to contain the mouse. Mice were timed from the moment its back feet first touched the plate until one or both feet left the plate (either from the mouse licking or jumping). Mice were removed from the plate if they had not lifted their feet after 30 s. For the tail-flick test, mice were placed head-first into a 50 ml tube (Falcon, BD, NJ, USA) for restraint purposes, with breathing holes drilled into the end and the mouse's tail and hind legs protruding. An automated tail flick apparatus (Harvard Instruments, MA, USA) was used and a high-intensity light was focused on the dorsal surface of the tail, approximately 15 mm from the tip. An infrared beam was used to determine when the mouse's tail moved and the latency for the tail to move was recorded for three consecutive trials for each mouse. Mice were removed from the light source if they had not moved their tails after 10 s.

### Statistical Analyses

All data were analysed using the SPSS software package (ver. 17, SPSS Inc., IL, USA). For the 5C-SRT and 5C-CPT, variables that were expressed as a fraction or rate (i.e. accuracy, omission rate, p(Hit) and p(FA) were subjected to arcsine transformation to limit the effect of an artificially-imposed ceiling [43]. For all tests, ANOVA were used to assess effects of Maternal diet, Sex and Pre-exposure (in the case of LI experiments), on the main dependent variables for each experiment. Repeated measures factors were used where appropriate (e.g. CS number for LI experiment, stimulus duration for multiple stimulus durations experiment). Significant main effects were followed by post hoc *t*-tests. Significance was set at *P*<0.05. Where no significant sex interactions were observed data were presented pooled for sex.
